# Recurrent midgut volvulus in an adult patient — The case for pexy? A case report and review of the literature

**DOI:** 10.1016/j.ijscr.2019.11.037

**Published:** 2019-11-27

**Authors:** Margarida S. Ferreira, Joana Simões, António Folgado, Sandra Carlos, Nuno Carvalho, Filipa Santos, Paulo Matos Costa

**Affiliations:** a Department of General Surgery - Hospital Garcia de Orta, Avenida Torrado da Silva, 2805-267 Almada, Portugal; b Faculdade de Medicina da Universidade de Lisboa, Avenida Professor Egas Moniz, 1649-028 Lisboa, Portugal

**Keywords:** Intestinal malrotation, Midgut volvulus, Recurrent, Case report, Ladd procedure

## Abstract

**Introduction:**

Intestinal malrotation is a rare condition, with an incidence estimated between 0,2 to 1%. Most cases are diagnosed and treated during childhood. Adult presentations are rare and most adults present with chronic nonspecific complaints.

Midgut volvulus is the most feared complication of intestinal malrotation, far more common among the pediatric than the adult population. Presentation in adulthood with a midgut volvulus accounts for a minority of these patients (15%).

The Ladd procedure is the standard surgical management of midgut volvulus and intestinal malrotation. Most evidence on the outcomes of the Ladd procedure originates from studies on pediatric population and the recurrence among children who have had a Ladd procedure is low (2–7%).

**Presentation of case:**

We report an exceedingly rare case of a patient who presented in adulthood with a midgut volvulus and less than two years after undergoing Ladd procedure presented with a recurrence of the midgut volvulus. The recurrent midgut volvulus was successfully treated by a fixation procedure (cecopexy and duodenopexy).

**Conclusion:**

Reports of midgut volvulus in adult patients are scarce and reports of recurrence even scarcer hence the rate of recurrence among adult patients has yet to be determined. The recurrence rate in some of the available adult series is much higher than the rate reported among children.

Should the rate of recurrence among adult patients prove higher, it poses the question of whether the Ladd procedure should be modified to include bowel fixation when performed in adults.

## Highlights

• Intestinal malrotation and midgut volvulus in adulthood are rare events.
• Reports of recurrence among adults are very scarce. The rate of recurrence and optimal surgical management are yet to be determined.
• Bowel fixation procedures may be considered in Ladd procedures for adult midgut volvulus in order to reduce recurrence.

## 1 Introduction

Intestinal malrotation arises from a default in the intestinal rotation around the superior mesenteric artery that takes place during the normal embryonic development.

It is a rare event with a reported incidence ranging from 1/6000 to 1/200 (0.5%) of live births and 1% in autopsy studies [1,2].

Congenital intestinal malrotation is commonly diagnosed during childhood: an estimated 90% of patients are diagnosed during the first year of life [1,2] and adult presentations are rare and reported to account for only 0.2-0.5% of intestinal malrotation cases [[3], [4], [5], [6]]. Adult presentations are most commonly either incidental findings on radiological studies or chronic non-specific complaints such as abdominal pain or intermittent episodes of bowel obstruction. Only a minority of adult malrotation cases present with midgut volvulus [[7], [8], [9], [10]].

Among patients presenting with chronic complaints, the episodes are typically recurrent bouts of abdominal pain, which may be associated with nausea, vomiting, constipation or diarrhea. Such bouts have often been present since childhood [[7], [8], [9], [10]].

In a cohort of 82 adult patients Nehra et al. reported the most common complaints to be abdominal pain (present in 87% of patients), emesis (present in 37% of patients) and nausea (present in 31% of patients). Although only an estimated 15% of adults with malrotation present with midgut volvulus, it is the most worrisome complication of intestinal malrotation: in the absence of timely diagnosis a midgut volvulus may have catastrophic consequences with progression to gangrene of the rotated gut, sepsis and the need for extensive small bowel resection with subsequent short bowel syndrome [1,3,7].

The surgical treatment of intestinal malrotation in children was described by Willian Ladd in 1936. The Ladd procedure consists of counter clockwise detorsion of the volvulus, division of all bands and adhesions securing the duodenum and bowel in abnormal position (the so-called ‘Ladd bands’), widening of the small bowel mesentery with repositioning of the duodenum and jejunum towards the right quadrant and repositioning of the colon towards the left side and appendectomy as the cecum will be positioned on the left [11].

Recurrence of midgut volvulus after a Ladd procedure during childhood is a rare event [12,13]. Given the rarity of adult presentations of midgut volvulus, information on prognosis and reports of recurrence after Ladd procedure in adulthood are even more scarce.

This work is reported according to the SCARE 2018 criteria [14].

## 2 Presentation of case

A 22-year-old black male patient was admitted to the emergency department for non-specific abdominal pain. The patient described a periumbilical pain, lasting 12 h, worsened by food ingestion and he denied having nausea, emesis or changes in bowel habit. On further questioning the patient reported multiple episodes during his childhood of recurrent abdominal pain, occasionally accompanied by self-limited periods of bowel obstruction.

The patient had no relevant personal or family history. On physical examination he showed no signs of dehydration, vital signs were normal and he had no fever. Abdomen was soft, there was periumbilical tenderness but no signs of peritonitis. Blood tests were unremarkable, with a normal blood cell count and normal C-reactive protein.

**Fig. 1:**
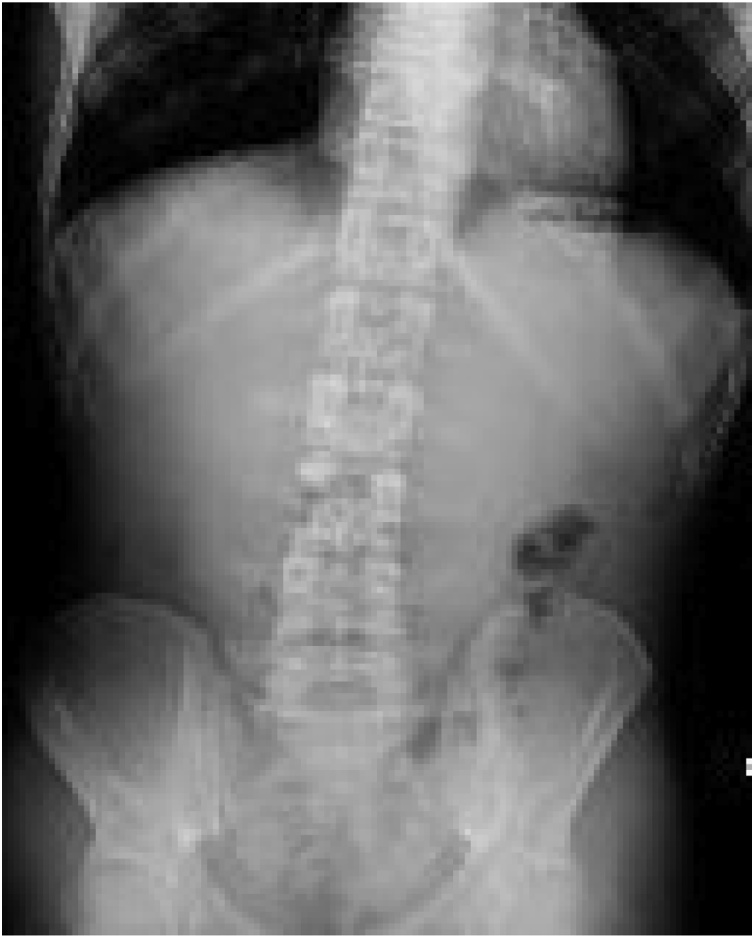
Abdominal x-ray unremarkable except for a decrease in bowel air content.

Abdominal x-ray was non-specific, showing a decrease in bowel air content (Fig. 1).

**Fig. 2:**
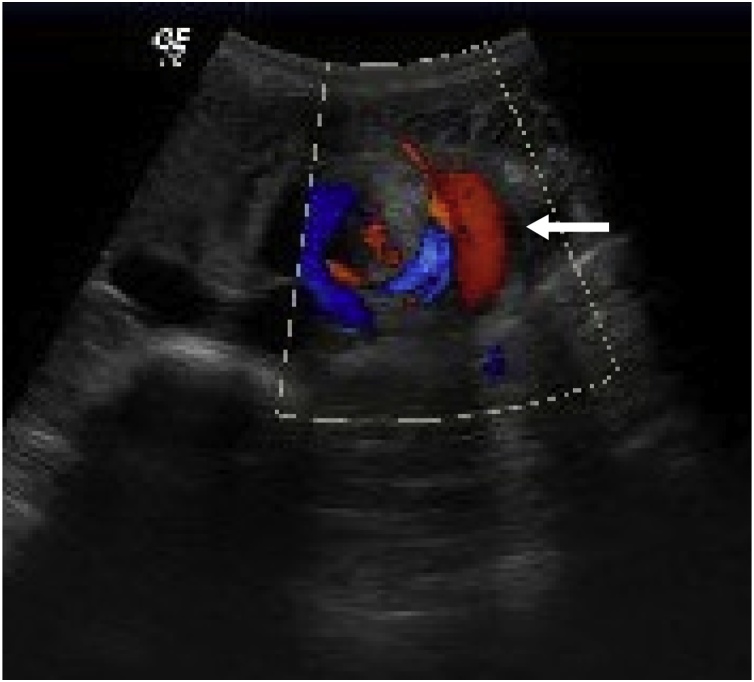
Abdominal ultrasound with whirlpool sign (white arrow) - hallmark of superior mesenteric vessels torsion.

Abdominal ultrasound showed the hallmark whirlpool sign [15] of superior mesenteric vessels torsion; marked gastric distention and no free fluid (Fig. 2).

**Fig. 3:**
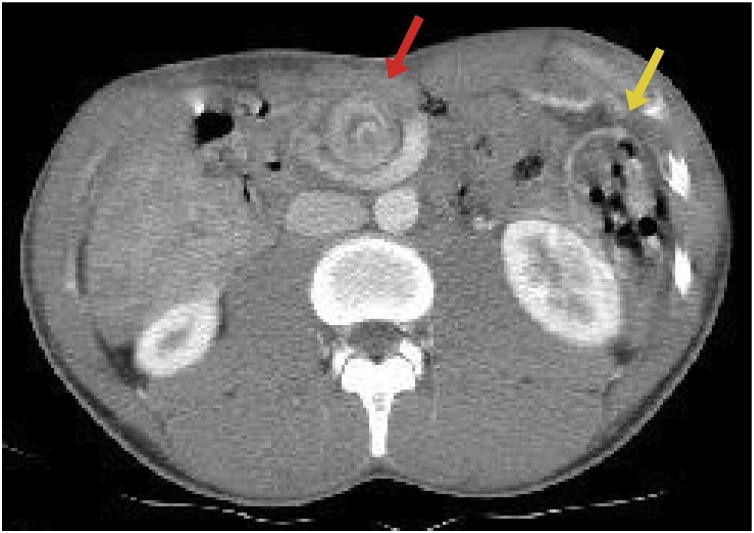
Abdominal CT scan showing aberrant bowel placement (yellow arrow) and mesenteric vessel torsion (red arrow).

A diagnosis of acute midgut volvulus was established and an intravenous contrast CT scan was performed for further characterization of the malrotation. The CT showed aberrant distribution of bowel loops with the colon located on the left side and small bowel loops on the right side of the abdomen and torsion and dilation of the superior mesenteric vessels (Fig. 3).

**Fig. 4:**
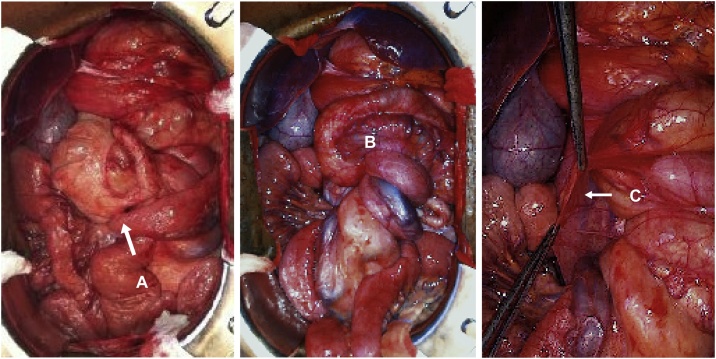
Intra-operative findings at laparotomy: abnormal positioning of the cecum (A); view of the midgut volvulus (B) and Ladd bands (C).

The patient underwent urgent laparotomy with intra-operative confirmation of midgut volvulus due to intestinal malrotation. A Ladd procedure was performed: detorsion of the volvulus, division of ‘Ladd bands’, widening of the small bowel mesentery, repositioning of the duodenum and jejunum towards the right quadrant and the colon towards the left side and appendectomy (Fig. 4A–C).

Post-operative course was uneventful.

Two years after the Ladd procedure the patient was re-admitted for acute abdominal pain with 48 h of duration, he denied nausea, emesis, or fever.

On physical examination the abdomen was distended, tympanic, bowel sounds were diminished and there was tenderness on palpation.

Blood tests were positive only for an increase in white blood cell count.

CT scan showed some free fluid, distended jejunal and ileal bowel loops and a collapsed colon with no identifiable transition point. There was no evidence of midgut volvulus recurrence.

Due to the worrisome findings of tenderness on physical examination, elevated white blood cell count and free fluid on the CT scan a decision was made to operate. The patient underwent a laparotomy and a recurrence of the midgut volvulus around the mesenteric axis was found. Following lysis of adhesions, the bowel rotation was reduced and a colopexy of the cecum to the left gutter was performed along with a pexy of the jejunum in the right gutter. Post-operative course was uneventful. At 3 years of follow-up since the second procedure, the patient has had no other events or symptoms.

## 3 Discussion

Recurrence of midgut volvulus after a childhood Ladd’s procedure is rare, reported to be between 2% and 7% [12,13,16].

Given the lower incidence of adult midgut volvulus (there are about 120 case reports and another 200 patients reported in 14 case series in the literature) the recurrence rate among adult patients is harder to estimate [3,[5], [6], [7], [8], [9], [10],[17], [18], [19], [20], [21], [22], [23], [24]].

Table 1 presents a review of the reported case series of adult Ladd procedures. The series are small and heterogeneous including both intestinal malrotation and midgut volvulus cases and they vary widely in follow-up period.

**Table 1:** Summary of the case series of adult Ladd procedures reported in the literature.

	Malrotation (MR) or Midgut volvulus (V)	Duration of follow-up	n adult Ladd procedures	n adult recurrences	% recurrence
Husberg et al. [17]	19 MR cases + 12 cases V	1–12 years	31 (26 at follow- up)	5	16%
Fu et al. [10]	7 MR cases + 4 cases “with obstruction”	2–118 months	11	2	18,0%
Kotobi et al. [18]	5 acute presentations	12–270 m (mean 63 m)	11	0	0%
Anand et al. [19]	20 MR + 2 V	8 years	22	0	0%
Zheng et al. [20]	Volvulus	3 m–5 y	8	0	0%
Prasad et al. [21]	MR	1–5 years	42	0	0%
Matzke et al. [8]	16 MR cases + 5 cases V	2 w– 97 m (mean 42 m)	21 (16 at follow- up)	0	0%
Seymour and Andersen [22]	4 MR cases + 3 cases V	2–48 m	7	0	0%
Durkin et al. [23]	8 MR cases + 2 unknown	–	9	0	0%
Nehra and Goldstein [7]	72 cases MR + 10 cases V	no follow-up	29 adult Ladd procedures	–	–
Von Flue et al. [6]	6 cases MR + 4 cases V	6 m–10 y	6 own series + 6 cases literature	0	Own series includes fixation in standard Ladd
Dietz et al. [9]	MR		4	0	0%
Moldrem et al. [5]	MR	no follow-up	33	–	–
Fukuya et al. [24]	Volvulus	no follow-up	5	–	–

MR = malrotation, V = midgut volvulus, w = weeks, m = months, y = years.

The best available evidence among children places the recurrence rate at 1.4%–3.5% (for open and laparoscopic Ladd procedure, respectively) [12,13,16,[25], [26], [27], [28]]. The rate of recurrence among patients who underwent Ladd’s procedure in adulthood in some of the series is much higher than that reported in children with a recurrence rate as high as 16% [17] and 18% [10] in some series.

To the best of our knowledge, ours is the 9th case to be reported in the literature of adult recurrence of midgut volvulus after Ladd’s procedure during adulthood. Sheik and Alkan’s cases were not included as their cases report recurrence in adults that had a Ladd procedure in childhood [[5], [6], [7], [8], [9], [10],[17], [18], [19], [20], [21], [22], [23], [24],^29^,^30^].

No high-level evidence is available to support management decisions on adult intestinal malrotation and volvulus. Moreover, it should be noted that while the literature significantly overlaps adult intestinal malrotation and midgut volvulus these are two different presentations of the same condition with different symptoms, severity and optimal management. It is questionable that data on adult intestinal malrotation should be the grounds for surgical management of adult midgut volvulus. The debate is still ongoing as to whether asymptomatic or minimally symptomatic (usually an incidental finding) intestinal malrotation warrants surgical treatment in adults [7,8,23,[31], [32], [33]].

The question of whether performing a fixation (pexy) of the abnormally positioned bowel segments diminishes the incidence of subsequent complications after Ladd procedure is long standing. Alexander Bill modified Ladd procedure in 1966 adding a suture of the posterior duodenum to the right renal fascia and a suture of the cecum to the left abdominal wall, arguing that this fixation would decrease recurrence of the midgut volvulus. In the 1980’s Stauffer [34] reported a series of 77 children who had underwent either a Ladd procedure or Ladd procedure plus fixation and found a trend for less reoperation in the group with pexy but no significant difference in the recurrence rate among the two groups [34,35].

Stauffer’s case series remains isolated as the highest quality available evidence on the matter, and while textbook and review authors [1,36] do not recommend a fixation procedure be added, a review of the available case series [6,24,33,37] shows that it is not uncommon practice to add a fixation procedure and it does constitute a safe option. Other options including the far more complex Fitzgerald and Wangensteen procedure have also been reported [38].

As among children the recurrence rate is low, the pexy may constitute an unnecessary procedure. It should perhaps be considered when performing Ladd procedure in adult patients where, with a low morbidity and mortality risk, it may have the potential to lower recurrence of midgut volvulus.

Durkin et al. had previously pointed out that the rate of reoperation increases the later a child is operated on [23].

One can hypothesize that adult patients, with a longer course of disease, may have more dramatic fibrotic changes in the mesentery making them more prone to a recurrence of the midgut volvulus around the mesenteric axis and it poses the question of whether additional gestures should be performed in the event of an adult Ladd procedure.

After recurrence of the midgut volvulus in our patient, we chose to perform a pexy of the small and large bowel in an attempt to avoid recurrence. At 3 years of follow-up since the last procedure, the patient’s course has been uneventful.

Further studies are necessary to establish the true incidence of recurrence of adult midgut volvulus and to define with a higher level of evidence its optimal management. The rarity of these cases may never allow for a definite answer though.

Another rather rare aspect of our case report worth emphasizing is that the patient initially presented acutely with a midgut volvulus and while emesis and intermittent abdominal obstruction would prompt the alert pediatric surgeon to consider midgut volvulus these symptoms may well be underrated in an adult patient and lead to a late diagnosis after bowel ischemia has settled in.

Finally, it should be noted that as with all patients with a personal history of laparotomy, small bowel adhesions are the most common cause of intestinal obstruction in patients who have previously undergone a Ladd procedure [26]. However, given the dramatic consequences of a missed diagnosis of recurrence of midgut volvulus, this diagnosis should be firmly excluded before non-operative management of small bowel adhesions is undertaken.

As with all cases of intestinal obstruction, tenderness on physical examination, lab results positive for systemic inflammation or persistence of symptoms after nasogastric tube decompression should result in a low threshold for operative management.

## 4 Conclusion

Midgut volvulus is a potentially catastrophic consequence of intestinal malrotation. With most cases diagnosed and surgically corrected during childhood, the incidental diagnosis of intestinal malrotation or the acute diagnosis of midgut volvulus in the adult are rare events.

The surgical management of midgut volvulus consists in the Ladd procedure. Recurrence of midgut volvulus among children who have had such procedure is low and the most common cause of intestinal obstruction among these patients is small bowel adhesions.

The low incidence of midgut volvulus among adults results in a lack of data on the outcomes of an adult Ladd procedure. While recurrence seems to be an exceedingly rare complication of an already rare procedure, the recurrence rate for adult Ladd procedure derived from the scarce available data suggests it may be higher than that reported among children. This finding raises the question of whether the Ladd procedure performed in adults should be modified to include bowel fixation.

## Sources of funding

This report did not receive any specific funding from public, commercial, or not-for-profit sectors.

## Ethical approval

Ethical approval has been exempted by our institution.

## Consent

Written informed consent was obtained from the patient for the publication of this case report and accompanying images. A copy of the written consent is available and can be reproduced whenever needed.

## Author’s contribution

MF, JS, AF, SC, NC and FS participated in the operations and perioperative management of the patients. MF acquired and interpreted date and drafted the manuscript. JS, AF, SC, NC, FS and PC contributed to manuscript drafting and revision. All authors read and approved the final manuscript.

## Registration of research studies

Not applicable.

## Guarantor

Margarida S. Ferreira.

## Provenance and peer review

Not commissioned, externally peer-reviewed.

## Declaration of Competing Interest

None of the authors has any conflict of interest to declare.
